# Confusion Assessment Protocol: Italian Cross-Cultural Adaptation and Validation

**DOI:** 10.3390/brainsci15101102

**Published:** 2025-10-13

**Authors:** Giulia Ferri, Anna Carannante, Manuela Iannetti, Sara Schiattone, Paola Ciurli, Fabiana Mogavero, Valentina Massimi, Marta Aloisi, Rita Formisano, Marco Giustini

**Affiliations:** 1 IRCCS Fondazione Santa Lucia, 00179 Rome, Italy; g.ferri@hsantalucia.it (G.F.); m.iannetti@hsantalucia.it (M.I.); s.schiattone@hsantalucia.it (S.S.); p.ciurli@hsantalucia.it (P.C.); fabiana.mogavero@uniroma1.it (F.M.); valentina.massimi@uniroma1.it (V.M.); r.formisano@hsantalucia.it (R.F.); 2 National Institute of Health, 00161 Rome, Italy; anna.carannante@iss.it (A.C.); marco.giustini@iss.it (M.G.)

**Keywords:** severe acquired brain injury (sABI), post-traumatic confusional state (PTCS), Confusion Assessment Protocol (CAP), neuropsychological assessment, cultural adaptation, psychometric validation

## Abstract

**Background**: This study validated the Italian version of the Confusion Assessment Protocol (CAP), a tool designed to assess Post-Traumatic Confusional State (PTCS) in patients with severe acquired brain injury (sABI) who are not evaluable with standard neuropsychological evaluations. **Objectives:** The primary aim of this study was to promote the CAP as a tool for assessing patients who are not still eligible for standard neuropsychological evaluation and to adapt it to Italian-speaking sABI patients by translating it into Italian and conducting a cross-cultural adaptation and evaluating its psychometric properties. The secondary objective was to correlate the CAP scores with broader functional scales, such as the Levels of Cognitive Functioning Assessment Scale (LCF) and Disability Rating Scale (DRS). **Methods**: A total of 42 sABI patients were enrolled at IRCCS Fondazione Santa Lucia. The CAP was translated and culturally adapted using international back-translation guidelines. Cross-cultural validity was assessed in 20 patients. The final version was administered by three trained raters over two days to evaluate inter- and intra-rater reliability. **Results**: The Italian version of the CAP demonstrated high internal consistency and substantial inter-rater reliability for key symptoms, including night-time sleep disturbances, decreased daytime arousal, and psychotic-type symptoms. Cognitive impairment showed moderate inter-rater agreement, likely due to symptom fluctuations typical of this recovery phase. The convergent validity of the CAP was confirmed through its correlations with the Levels of Cognitive Functioning (LCF) and the Disability Rating Scale (DRS), demonstrating its clinical utility in integrating cognitive and behavioral symptom assessments. **Conclusions:** The Italian version of the CAP is a reliable and valid tool for assessing PTCS in sABI. Future developments should address limitations related to symptom intensity, behavioral domains, and differential symptom weighting.

## 1. Introduction

### Severe Acquired Brain Injury (sABI) and Disorders of Consciousness (DoC)

Patients recovering from severe acquired brain injury (sABI) often evolve through a series of clinical states. A well-established nomenclature describes the various clinical conditions that can emerge during this recovery process, including coma, vegetative state (VS) (also termed as unresponsive wakefulness syndrome, UWS) [1], and minimally conscious state (MCS) [2,3]. Conversely, the clinical condition observed when patients evolve from MCS to a higher level of consciousness, is less clearly defined and has been labeled in various ways, including emerged from MCS (E-MCS), post-traumatic amnesia (PTA) [4], traumatic delirium [5], and post-traumatic confusional state (PTCS) [6].

Patients with E-MCS demonstrate increased responsiveness and intentional communication compared to those in MCS (e.g., through the presence of functional and appropriate communication and/or functional use of an object), but their self-awareness remains severely impaired. During this recovery phase, patients may exhibit confusion despite being conscious and responsive. Descriptions of these clinical states highlighted a wide range of deficits, including agitation, irritability, disorientation, impaired perceptual abilities, decreased judgment, memory and language impairment, decreased level of arousal, disinhibition, and inappropriate mood [7,8]. Acute confusion is common in patients with sABI admitted to inpatient rehabilitation, often leading to challenges in patient management and safety issues for both patients and staff and also for family members who no longer recognize their relatives and do not know how to relate to them.

Some clinicians and researchers refer to this clinical state as PTA, a term coined by Russell (7). PTA is characterized by a range of neurobehavioral symptoms [7,8], including disorientation for time, place, and situation, as well as impairments in recognition memory. Tools such as the Galveston Orientation and Amnesia Test (GOAT) and the Westmead Post-traumatic Amnesia Scale are widely used to assess these symptoms [9,10,11].

Indeed, PTA may be considered a subset of PTCS, since the memory deficit is only one of the features of a more complex confusional state.

However, the heterogeneity of these patients’ profiles makes it necessary to standardize the terms, definitions, and clarify the diagnostic criteria, aiming to improve both clinical management and research consistency in this area.

Stuss et al. [12] proposed the term Post-Traumatic Confusional State (PTCS) to describe this phase of recovery. PTCS was defined as “a transient organic mental syndrome with acute onset characterized by a global impairment of cognitive functions with a concurrent disturbance of consciousness, attentional abnormalities, reduced or increased psychomotor activity, and a disrupted sleep/wake cycle.” [12]. This term offers a more comprehensive description than PTA, emphasizing attention deficits as the primary cognitive impairment, rather than solely anterograde amnesia.

This “transient organic mental syndrome” may be due to several neurobiological bases, such as diffuse axonal injury, frontal lobe dysfunction, cerebral edema, and disconnection syndrome [13,14,15,16].

Zafonte et al. [17] have demonstrated that the duration of PTA is a significant predictor of functional outcome after ABI. This underlines the predictive value of disorientation severity and duration for recovery outcomes.

For this reason, it is crucial to include a range of neurobehavioral phenomena observed during the early recovery stages after sABI in order to conduct a thorough preliminary assessment. Sherer’s research extends the assessment of PTCS to core and associated features evaluated using various established clinical tools. However, instead of relying on separate tests for each symptom, it is more effective to use comprehensive assessments that evaluate multiple dimensions of PTCS simultaneously. These tools should cover both core and associated symptoms, track their severity, and provide criteria for defining the boundaries of PTCS.

To ensure accurate assessment of all features of PTCS, it is essential that measurement tools are validated. Sherer et al. [18] introduced the Confusion Assessment Protocol (CAP) as one of the most comprehensive tools for assessing PTCS.

An international team of researchers and clinicians (known as INCOG) met to update the INCOG 2.0 Guidelines [19] that defined PTCS as a clinical condition characterized by four core features: (i) disturbances of attention: reduced ability to focus or sustain attention; (ii) disorientation: impaired orientation to place, time, and situation; (iii) disturbances of memory: impaired ability to encode and recall new information; (iv) fluctuation: the character and severity of the disturbances varies throughout the day.

This latter classification may be of clinical relevance, since it supports the diagnostic process of this complex confusional state.

In addition to these core features, patients with PTCS may also experience emotional problems, behavioral dysregulation, sleep–wake cycle dysregulation, delusions, perceptual disturbances, and confabulation.

The impairment profiles of these patients are highly variable, affecting neuromotor, cognitive, emotional, behavioral, and neurofunctional domains. One of the main objectives in developing a case definition for PTCS was to improve diagnostic accuracy and reliability in both clinical and research settings.

Based partially on the Delirium Rating Scale-Revised 98 (DRS-98) [20] and other standardized measures, the CAP has been proposed to capture a wide range of symptoms associated with PTCS. It defines the presence or absence of PTCS by classifying patients as confused if they exhibit four or more symptoms, or three symptoms including disorientation. Additionally, the CAP provides an index of PTCS severity, classifying confusion as mild if three or four symptoms are present, moderate with five symptoms, and severe if six or seven symptoms are present. Therefore, this tool offers a brief and efficient method for assessing confusion symptoms, duration, and severity of different patterns, emergence from the confusional state, and potential stability of the condition.

The primary aim of this study is to introduce the CAP as a tool for assessing patients who have emerged from a minimally conscious state but are still not eligible for standard neuropsychological evaluation. Specifically, we aimed to adapt the CAP for Italian-speaking persons with sABI by translating it into Italian, conducting a cross-cultural adaptation and evaluating its psychometric properties. As a secondary aim, the study correlates the CAP score with the Levels of Cognitive Functioning Assessment Scale (LCF) [21] and with the Disability Rating Scale (DRS) [22], a tool regularly used in clinical practice to assess cognitive functioning in patients in the early stages of the post-coma state. The CAP aims to improve diagnostic accuracy, facilitating both clinical practice and research; it will be evaluated for its reliability and consistency, compared to other observational scales used.

## 2. Materials and Methods

### 2.1. Population

Consistent with previous studies on the CAP [23] and according to the recommendations available in the literature, a minimum sample size of 20 patients was considered to evaluate the cross-cultural validity of the tool. In the subsequent validation phase, the final Italian version of the CAP was administered to 42 sABI patients admitted at the IRCCS Fondazione Santa Lucia in Rome, between May 2022 and April 2024, according to the criteria mentioned below. The sample size of 20 patients considered to evaluate the cross-cultural validity were included in the total number of patients enrolled.

The behavioral evidence of confusion was based on clinical judgment using DRS and LCF.

The inclusion criteria were as follows: (i) age between 15 and 70 years; (ii) diagnosis of severe acquired brain injury (sABI) with a coma duration of at least 24 h and a Glasgow Coma Scale [24] score ≤ 8; (iii) Level of Cognitive Functioning (LCF) [21] score between 4 and 6; (iv) emergence from the minimally conscious state (E-MCS) as recorded by the Coma Recovery Scale-Revised (CRS-R) [25,26]; (v) behavioral evidence of confusion such as disorientation, severe distractibility, and agitation, according to the scores of the DRS and LCF scales as determined by a neuropsychologist with long-term expertise in sABI. Patients who exhibited the following characteristics were excluded: (i) substance abuse or pre-existing neurological and/or psychiatric disorders, either stabilized or progressive; (ii) medical complications, infections, or other comorbidities which compromised the behavioral assessment; (iii) linguistic barrier; (iv) severe aphasia. In addition, medications acting on the central nervous system taken within 2 h prior to the administration of the CAP have been recorded.

The final sample comprised 42 patients: 31 males (73.8%) and 11 females (26.2%), with mean age 52.0 years (SD ±15.2) and mean educational level 12.4 years (SD ±3.7). All participants were included in the analyses without gender-based stratification, as gender was not a primary variable in this validation study. Complete demographic and clinical characteristics are presented in Table 1.

The study was approved by the local Ethics Committee of IRCCS Fondazione Santa Lucia, Rome, Italy (Protocol number: CE/2022 005) and performed according to the ethical principles introduced in 1964 by the Declaration of Helsinki and its later amendments (2014) [27]. The main caregivers of the patients enrolled signed an informed consent.

### 2.2. Translation and Cultural Adaptation

The original CAP was translated from English to Italian using international guidelines [28]. The first stage in the adaptation included forward translation according to the back-translation procedure. The original English version of the CAP was translated blindly into Italian by two evaluators familiar with English, who produced two independent literal translations. After that, two English native speakers—having not seen the original version—used the temporary version of the questionnaire to translate it back into the original language. The back-translated version of the tool was then compared to the original. To adapt the translated version to Italian culture, one neuropsychologist and two speech therapists, who were familiar with both English and Italian, reviewed the first translated version and then reworded it according to the Italian culture. As examples, in the Test Completion Codes we changed, in the Italian version: n.1: “arousal difficulty” as “impossibility to remain awake” instead of “stay awake”; n.5: “aphasia” as “severe damage” instead of “profound damage”; and finally in the comprehension test we changed all the questions of Form 1 and Form 2 to the present verbal time in Italian instead of the future one in English.

### 2.3. Pre-Test (Cross-Cultural Validity)

The reliability and validity of the culturally adapted scale was assessed by using the checklist titled “COnsensus-based Standards for the selection of health Measurement Instruments” (COSMIN) [29]. The prefinal translated version of the CAP was administered to a subset of patients (n.20) to evaluate its cross-cultural validity. To avoid bias, each patient was tested twice by the same evaluator. The time interval between repeated administrations was short enough to ensure that no clinical change had occurred, and a time period of 24 h was considered appropriate. This resulted in the final Italian version of the CAP being applied to the whole population of the study.

### 2.4. Validation

The final Italian version of the CAP was administered by three trained expert raters, speech therapists with at least 10 years of expertise in cognitive–behavioral treatment of patients with sABI.

The clinical neuropsychologist selected the patients according to the inclusion and exclusion criteria.

Each selected patient was assessed on two consecutive days.

Day 1: Within a 6 h time frame, two assessments were conducted on the same patient through the administration of the CAP by two random blinded raters (A and B), to evaluate inter-rater reliability. The order of administration was randomly selected.

Day 2: Rater A administered the CAP again to the same patient of the day before to evaluate intra-rater reliability.

The raters were familiar with the CAP prior to the study and patient’s clinical status (arousal level, medical stability, etc.) was monitored to ensure stability across the two-day testing period.

In parallel and in a blind manner, the neuropsychologist completed the clinical scales included in the protocol (DRS and LCF).

The external scales were used only for validation purposes.

According to Sherer [23], through the administration of the CAP, seven key symptoms of post-traumatic confusional state were investigated: (1) cognitive impairment, (2) disorientation, (3) agitation, (4) fluctuation in symptom severity, (5) night-time sleep disturbance, (6) daytime decreased arousal, and (7) psychotic-type symptoms. Cognitive impairment was assessed with Toronto Test of Acute Recovery After TBI (TOTART) [12] and Cognitive Test of Delirium (CTD) [30]; scores ≤18 indicate substantial impairment and count as 1 symptom of post-traumatic confusion. Disorientation was assessed with the Galveston Orientation and Amnesia Test (GOAT) [11]; GOAT error scores >24 indicate disorientation and count as 1 symptom of post-traumatic confusion. Agitation was assessed with the Agitated Behavior Scale (ABS) [31]; ABS scores >17 indicate increased restlessness and count as 1 symptom of post-traumatic confusion. Fluctuation of symptoms, night-time sleep disturbance, decreased daytime arousal severity, and psychotic-type symptoms were assessed with the DRS-98 [20]:

- Fluctuation in symptom severity is coded as “present” if symptom intensity is rated as “fluctuating over hours or minutes”.
- Night-time sleep disturbance severity is coded as “present” if symptom intensity is rated as “moderate or severe disruption of the sleep–wake cycle”.
- Decreased daytime arousal is coded as “present” if symptoms intensity is rated as “patient has difficulty staying awake and alert during exam and therapy sessions or patient is unable to stay awake and alert”.
- Psychotic-type symptoms are coded as “present” if (i) perceptual disturbance including illusions or hallucinations; (ii) if patient is suspicious, has unusual ideation, or is delusional; (iii) or if thought processes are tangential or show loose associations.

A total CAP score was calculated by classifying all patients according to the number of symptoms of confusion. Namely, patients with 4 or more symptoms were confused or, if 1 of the symptoms was disorientation; patients with 3 or more symptoms were also considered confused. The thresholds for interpretation of the CAP score were as follows: (i) ≤3 symptoms: confusion was diagnosed as absent; (ii) 4 symptoms or 3 symptoms if 1 of the symptoms was disorientation: mild confusion; (iii) 5 symptoms: moderate confusion; (iv) 6/7 symptoms: severe confusion.

### 2.5. Statistical Analysis

The sample size of 42 participants was considered adequate for the statistical techniques employed in this study. According to the methodological literature, samples of about 30–50 participants are generally sufficient to obtain stable estimates of inter-rater agreement (Cohen’s kappa), internal consistency coefficients for dichotomous items (KR-20), and correlation coefficients (Spearman’s rho) with acceptable precision [32,33,34]. To assess the internal consistency reliability of CAP’s key symptoms that have dichotomous (binary) items, the Kuder–Richardson coefficient of reliability (KR-20) was performed. Data provided include item difficulty, item variance, item–rest correlation for various key symptoms rated by three evaluators (A, B, A2), and overall KR-20 reliability coefficient. An overall KR-20 ≥ 0.61 was considered acceptable [35]. For the purposes of this study, item difficulty (*p*) is to be defined as the proportion of patients who have the key symptoms. The item variance in the KR-20 test measures the variability in response to each item on the test and is defined as *p* * (1 − *p*). Items with variances close to 0.25 (hence, item difficulty close to 0.50) were highly desirable for increasing test reliability. It is also important to have a range of item difficulties to cover the entire ability spectrum of the test-takers. Therefore, a well-constructed test will include items with a variety of item difficulty values, not just those near 0.5. Generally, the recommended item variance values range between 0.3 and 0.7 to maximize test information and differences among the examinees [36]. Item–rest correlation refers to the correlation between the score on an individual item and the total score on the rest of the test (excluding the item in question). This metric is used to assess how well each item correlates with the overall test score, which is indicative of the item’s consistency with the other items in measuring the same construct. As a rule of thumb, values between 0.20 and 0.39 indicate good discrimination, while values 0.4 and above indicate very good discrimination [37,38].

For each of the seven CAP key symptoms and for the CAP score, Cohen’s Kappa (k) was carried out to assess the inter-rater and the intra-rater reliability in the classification of patients. Unweighted Cohen’s kappa was used for all analyses, in line with the original CAP methodology [18]. Inter-rater reliability refers to the extent to which two raters (A vs. B) give concordant assessments of the same patient. This measure indicated how much homogeneity there was among different raters. Intra-rater reliability refers to the consistency of ratings made by the same rater across two evaluations (A vs. A2). This measure indicated how stable a rater’s different assessments in the same period was over time. The k statistics was interpreted as follows: values ≤ 0 indicate no agreement, 0.01–0.20 indicate none to slight, 0.21–0.40 indicate fair, 0.41–0.60 indicate moderate, 0.61–0.80 indicate substantial, and 0.81–1.00 indicate an almost perfect agreement [39].

The Spearman rank correlation coefficient, defined as ρ (rho), was used to assess the strength of the relationship between the three CAP scores obtained by the three raters’ administrations, LCF and DRS scores. We assumed that the correlation was (a) very high if ρ ≥ 0.90; (b) high if 70 ≤ ρ < 90; (c) moderate if 50 ≤ ρ < 70; (d) low if 30 ≤ ρ < 50; (e) negligible if 0 ≤ ρ < 30 [34].

Cramér’s V was calculated to derive the effect size measurement for the chi-square test of independence between each couple of key symptoms. As each patient was evaluated three times (by raters A, B, and A2), Cramér’s V was computed separately for each rater’s dataset and then averaged across the three administrations to obtain a single summary measure per symptom pair. This approach was adopted because the analysis was intended to be exploratory, aimed at identifying potential symptom clusters rather than testing specific hypotheses about symptom associations. Given the sample size (*n* = 42), more complex repeated-measures frameworks (e.g., GEE or mixed-effects models) were considered unlikely to yield stable estimates for all 21 symptom pairs examined. Averaging the three coefficients reduced noise from rater-specific variability while providing concise and interpretable summary measures. We acknowledge that this is a simplified approach that does not account for the correlation structure between repeated measures, and we have considered this limitation in the interpretation of results. For the interpretation of the effect size, the following thresholds were used: range values of 0.00–0.10 were considered as negligible; range values of 0.10–0.20 were weak; range values of 0.20–0.40 were considered moderate; range values of 0.40–0.60 were relatively strong; range values of 0.60–0.80 and 0.80–1.00 were considered strong and very strong, respectively [40].

The Clopper–Pearson “exact” method for calculating binomial confidence intervals was used [41]. Any *p*-value < 0.05 was considered as statistically significant. Statistical analysis was performed using STATA Stata/SE 15.1 (StataCorp, College Station, TX, USA).

## 3. Results

### 3.1. Descriptive Statistics

Data were collected for 42 patients consecutively enrolled for 2 years (May 2022–April 2024). All patients enrolled had a mean age of 52.0 years (SD: ±15.2 years) and a mean of educational years equal to 12.4 years (SD ±3.7); the majority were male (73.8%). All but one of the patients had severe TBI with a GCS score ≤ 8 in the acute phase. The mean interval from injury onset to the date of CAP administration (chronicity) was 69.3 days (SD: ±82.9 days). In 54.8% of patients, coma length, defined as time to follow commands, ranged from 1 to 14 days, in 38.1% from 15 to 30 days, in 7.1% lasted more than 30 days, with an overall mean coma duration of 15.5 days (SD: ±11.5 days).

Regarding the etiology of sABI, 26 out of 42 patients (61.9%) consisted of TBI, whilst 16 (30.1%) were classified as non-TBI due to hemorrhage, ischemia, and subarachnoid hemorrhage or cerebral anoxia. At CAP administration time point, patients had LCF and DRS scores with median values equal to 5 and 15, respectively (Table 1).

### 3.2. Internal Consistency (KR-20 Analysis)

According to the KR-20 analysis, the item difficulty values ranged from 0.24 to 0.90 across key symptoms. Cognitive impairment, disorientation, agitation, and fluctuation in symptom severity showed higher item difficulty values (0.69–0.90; mean 0.82), indicating that these symptoms were frequently observed. In contrast, night-time sleep disturbance and psychotic-type symptoms showed lower difficulty values (0.38–0.48), and daytime decreased arousal had the lowest difficulty (0.24), indicating that these symptoms were less frequently observed (Table 2). For the key symptom cognitive impairment and fluctuation in symptom severity, the variance is relatively low (0.16 and 0.18 on average between raters), indicating a good uniformity of the scores. The variance is even lower, consistent with the high difficulty values, for “disorientation” and “agitation” (0.13 and 0.9 on average between raters, respectively). On the contrary, for the key symptoms “night-time sleep disturbance” and “psychotic-type symptoms”, the variance is higher, indicating greater variability in responses, whereas the variance is moderate for “daytime decreased arousal”, consistent with the moderate difficulty of the items (Table 2).

All the key symptoms show item–rest correlations values ≥0.20, suggesting at least a good consistency with the overall test. “Night-time sleep disturbance” and “daytime decreased arousal” show very high item–rest correlation values (0.55 and 0.52 on average between raters, respectively), suggesting that these key symptoms were well aligned with the test, with a very good discrimination power.

As shown in Table 2, the KR-20 coefficient is 0.84, which was quite high and indicates that the test has good internal consistency, suggesting that the items were well correlated with each other and consistently measure the same construct.

**Figure 1:**
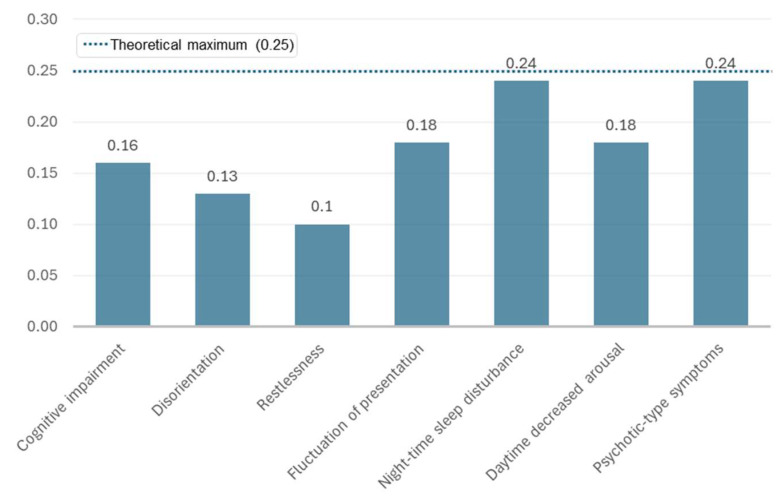
Average item variance (*p*×(1 − *p*)) for each of the seven CAP key symptoms across the three raters (A, B, A2).

As shown in Figure 1, night-time sleep disturbance and psychotic-type symptoms showed the highest average item variance (~0.24), indicating greater response variability among raters. In contrast, restlessness and disorientation showed the lowest variance values (~0.10–0.13), consistent with their high prevalence. This visual representation complements the detailed values reported in Table 2 and facilitates interpretation of item difficulty and variance patterns.

### 3.3. Inter- and Intra-Rater Reliability (Cohen’s Kappa Analysis)

Table 3 shows the Cohen’s k for the inter-rater and intra-rater reliability. Regarding the inter-rater reliability (same day, different raters), moderate agreement was observed for “cognitive impairment” key symptom (k = 0.41); substantial agreement was obtained for “disorientation” (k = 0.77), “agitation” (k = 0.63), fluctuation in symptom severity (k = 0.72) key symptoms. On the other hand, almost perfect agreement between raters was recorded for “night-time sleep disturbance” (k = 0.90), “daytime decreased arousal” (k = 0.94) and “psychotic-type symptoms” (k = 0.81). The CAP scores show substantial agreement between raters (k = 0.76).

Regarding the Intra-rater reliability (same rater, different days), moderate agreement between days of assessment was observed for “cognitive impairment” (k = 0.49), “disorientation” (k = 0.55), and “agitation” (k = 0.55) key symptoms. Substantial agreement was seen for fluctuation symptom severity (k = 0.70), “night-time sleep disturbance” (k = 0.66), “daytime decreased arousal” (k = 0.74), and “psychotic-type symptoms” (k = 0.75) key symptoms. As for the intra-rater memory effects, the fluctuations of the cognitive deficits, also in a short time window, may have played some role.

The intra-rater reliability, for the CAP scores, shows moderate agreement between the days of assessment (k = 0.45). Agreement analysis shows that there is generally greater reproducibility of results in assessments performed on the same day.

### 3.4. Convergent Validity (Correlations with LCF and DRS)

As shown in Table 4, there was a strong positive correlation between the CAP scores assessed by raters on the same day (ρ = 0.90), indicating that the two evaluators’ scores were highly concordant. There was a substantial positive correlation between the CAP scores assessed by the same rater on different days (ρ = 0.64), suggesting some variability in the rater’s scoring over time but still with a substantial level of consistency. In summary, the CAP scores showed significant correlations across different raters and different days, with the highest concordance observed between different raters on the same day. This suggests that while individual rater consistency over time was good, the inter-evaluator consistency on the same day was even better. The CAP correlates with the LCF score (ρ between −0.46 and −0.55) and with the DRS (ρ between 0.22 and 0.33).

For all three assessments (A, B, A2), there was no statistically significant association between CAP Score and LCF, although for assessment A2, an association on the edge of statistical significance was observed (*p* = 0.054).

### 3.5. Symptom Association Analysis (Cramér’s V Analysis)

According to Cramér’s V, relatively strong association was observed between “night-time sleep disturbance” with “daytime decreased arousal” (V = 0.46) key symptoms, while moderate association was observed between “night-time sleep disturbance” and “psychotic-type symptoms” (V = 0.37), “cognitive impairment” and “disorientation” (V = 0.32), “disorientation” and “fluctuation of symptom severity” (V = 0.29), “disorientation” and “restlessness” (V = 0.25), “daytime decreased arousal” and “psychotic-type symptoms” (V = 0.23), “restlessness” and “fluctuation of symptom severity” (V = 0.22), and “agitation” and “night-time sleep disturbance” (V = 0.21). The remaining values indicate weak or negligible associations, below V = 0.20 (Table 5).

### 3.6. Relationship Between CAP and LCF

Stratifying the distribution of key symptoms by LCF categorization, no statistically significant differences, in the proportion of symptoms in the two groups were observed except for “disorientation” (83.3% vs. 40.0%, *p* = 0.012) and “daytime decreased arousal” (33.3% vs. 0.0%, *p* = 0.037) key symptoms, which were much more frequent among the most severely disabled patients. However, the latter key symptom was missing among patients with LCF = 6 (Table 6).

Table 7 shows the comparison of the scores obtained, by the evaluators, using the two scales, CAP and LCF. Considering the three assessments of the 42 patients (rater A, B, A2), inconsistent grading occurred only three times out of a total of 126 ratings (2.4%), one involved a patient with a non-confused CAP and LCF = 4, and two consisted of patients with severely impaired CAP and LCF = 6.

## 4. Discussion

This study developed and validated the Italian version of the CAP, a tool designed to assess PTCS in patients with sABI who are still unable to undergo standard neuropsychological evaluations. The translation and cultural adaptation followed international guidelines, ensuring both semantic and conceptual equivalence under expert supervision.

To our knowledge, this is the first Italian validation of a multidimensional, clinician-rated tool specifically designed to capture the full PTCS symptom profile.

The reliability and validity of the Italian CAP were assessed using the COSMIN checklist.

The Italian CAP demonstrated strong psychometric properties, with high internal consistency (KR-20 = 0.84) and substantial inter-rater reliability (Cohen’s kappa = 0.63–0.94) for key symptoms, including “night-time sleep disturbances”, “daytime decreased arousal”, and “psychotic-type symptoms”. These findings confirm its reliability for clinical use. However, “cognitive impairment” showed only moderate inter-rater agreement (k = 0.41), which may be due to the complexity and variability inherent in this stage, characterized by symptomatic fluctuations and influence from the effects of pharmacological therapy. Intra-rater reliability ranged from moderate to substantial (k = 0.45–0.75), reflecting the CAP’s consistency over time, despite the inherent variability of PTCS symptoms.

The higher inter-rater reliability observed for same-day assessments (κ = 0.63–0.94) compared to intra-rater reliability across different days (κ = 0.45–0.75) likely reflects the clinical characteristics of PTCS rather than rater inconsistency. PTCS is inherently characterized by symptom fluctuation, which is recognized as one of its core diagnostic features. Consequently, greater variability across days is expected as part of the natural recovery trajectory, whereas within-day fluctuations are generally smaller and assessments performed within a few hours tend to capture a more stable clinical profile. This pattern suggests that lower agreement between different-day evaluations is more attributable to true symptom variability than to rater error. From a clinical perspective, this highlights that same-day assessments by different raters may provide a more reliable snapshot of a patient’s status, while longitudinal monitoring should account for the expected day-to-day variability of PTCS symptoms.

Cramer’s V analysis revealed two clinically relevant symptom clusters: one including “daytime decreased arousal”, “night-time sleep disturbances”, and “psychotic-type symptoms”, and the other involving “cognitive impairment”, “disorientation”, “agitation”, and “symptom fluctuation”. These interrelations, partially consistent with prior studies by Sherer et al., confirm the division of symptoms into primary and associated, reflecting, although not entirely adhering to, the classification proposed by Sherer. This evaluation helps clinicians to make more targeted interventions by addressing specific symptom clusters, optimizing treatment, and potentially improving patient outcomes.

The relationships between the CAP and other scales, such as the Levels of Cognitive Functioning (LCF) and the Disability Rating Scale (DRS), further demonstrate its clinical utility. CAP scores showed moderate negative correlations with LCF (ρ = −0.46 to −0.55), indicating that higher levels of confusion are associated with lower cognitive functioning.

This association, although moderate, has important clinical implications. The moderate negative correlation observed between CAP and LCF (ρ ≈ –0.5) indicates that higher levels of confusional symptoms are generally associated with lower levels of cognitive functioning, accounting for about 25% of shared variance. This magnitude supports the construct validity of CAP, while also confirming that it captures clinical aspects not fully reflected in LCF scores. From a clinical perspective, this suggests that CAP provides complementary information to LCF: while LCF offers a global index of post-coma recovery, CAP specifically profiles confusional symptoms, and the combined use of both measures may enhance clinical decision-making and treatment planning.

In our study, we observed cases where there was no consistency between the results of the two scales. Taken together, considering the three ratings of the 42 patients (rater A, B, A2), inconsistent grading occurred only three times out of a total of 126 ratings (2.4%), one involved a patient with “not confused” CAP and LCF = 4, and two involved patients with severe CAP and LCF = 6. This mild inconsistency may be due to the CAP intrinsic characteristic of the tool, based on dichotomous ratings for each key symptom (presence/absence) and the lack of intensity assessment. Indeed, according to the CAP, having three severe key symptoms is a less problematic situation than having six key symptoms observable enough to be detected.

The weak to moderate positive correlations found with the DRS suggests that the highest CAP scores are associated with the highest DRS scores, though the relationships were not strong. The CAP captures unique aspects of PTCS that are not fully addressed by the other disability scales. Consequently, the CAP complements these functional measures by offering detailed symptom tracking, enabling clinicians to more effectively align behavioral and cognitive interventions with patients’ needs.

The CAP’s ability to track specific symptoms and distinguish between mild, moderate, and severe PTCS further supports its integration into diagnostic and rehabilitative pathways. Although the CAP records only the presence or absence of individual key symptoms, it could be possible to graduate the intensity of the sub-scores into mild, moderate, and severe, adding the scores of the presence of symptoms according to the three different levels of severity.

Additionally, its sensitivity to symptom fluctuations enables monitoring of the effects of pharmacological and therapeutic interventions, facilitating timely adjustments to treatment plans.

Nonetheless, the CAP has several limitations, which may explain the weak correlation between CAP and LCF. First, it does not explicitly evaluate certain behavioral symptoms, such as inertia, unawareness, or confabulation. This assessment limitation could lead to an underestimation of the severity of behavioral deficits or imprecise evaluations in some cases. For instance, in patients with high levels of inertia or confabulatory behavior, the CAP might not reflect the true degree of impairment. This limitation may become more relevant in cases where these symptoms contribute significantly to the patient’s functional difficulties. Although previous validation studies of the original English CAP did not explore the possible different symptom weighting, further analysis on the possible predictive role of some symptoms on poor outcomes should be mandatory.

Future adaptations of the CAP could incorporate a broader assessment of behavioral symptoms to improve its comprehensiveness.

Furthermore, the CAP’s dichotomous scoring (presence/absence) does not assess the intensity of symptoms, which may lead to occasional discrepancies with other scales, such as the LCF. The CAP estimates the overall severity of the confusional state but does not capture the intensity of individual key symptoms, as it records only their presence or absence. As a result, severity is determined solely by the number of symptoms, without weighting their clinical relevance. This limitation may explain observed inconsistencies: for instance, three highly severe symptoms may be rated as less critical than six barely detectable ones.

This could oversimplify complex clinical presentations, leading to errors in the diagnostic classification of patients, since primary and associated symptoms contribute differently to the overall severity of PTCS. Future versions of the CAP could introduce a graded scoring system to account for the differential impact of each symptom on the overall clinical picture. This would enhance the CAP’s ability to capture the complexity of PTCS and provide more accurate insights into the patient’s condition.

Finally, a potential limitation of our intra-rater reliability design is the possibility of memory effects, as the same rater reassessed patients within a 24 h interval. Although this risk was mitigated by the fluctuating nature of PTCS symptoms, the complexity of CAP observations, and the experience of trained raters, some inflation of intra-rater reliability estimates cannot be excluded. Future studies could address this limitation by using longer retest intervals or employing different raters for temporal reliability assessment.

In summary, while the CAP is a reliable and valid tool for assessing PTCS in sABI patients, future research should address these issues. The introduction of more detailed measures for behavioral symptoms, symptom intensity and characterization, and differential weighting of different deficits would improve the CAP’s diagnostic efficacy and clinical utility.

## 5. Conclusions

The Italian CAP is a reliable and valid tool for assessing PTCS in sABI patients, offering detailed symptom monitoring and supporting individualized treatment planning. Its alignment with international standards and ability to complement disability scales make it a valuable addition to neuro-rehabilitation practices in Italy. Future research should aim to predict long-term outcomes, such as cognitive recovery and functional independence. Incorporating improvements in symptom evaluation could further enhance the tool’s clinical value and ensure its widespread applicability in diverse clinical settings.

Furthermore, the CAP aids in understanding which symptoms are more likely to co-occur, enhancing clinical assessments and enabling targeted interventions. Some symptom clusters may be clinically relevant; indeed, they could represent different subtypes of PTCS and guide intervention strategies. Identifying potential symptom clusters can provide valuable insights for diagnosis and treatment planning. Overall, these results reinforce the importance of using multiple reliable assessment tools in clinical practice to capture a holistic view of cognitive and behavioral deficits in patients with sABI in post-acute rehabilitation phase.

In conclusion, CAP may be a helpful part of the rehabilitation pathway to support decision-making on further therapeutic approach and/or long-term care of outpatient follow-up.

## Figures and Tables

**Table 1:** Socio-demographic and clinical aspects.

Male Gender (N/%)	31 (73.8%)
Age (years, mean/sd)	52.0 (±15.2)
Educational years (years, mean/sd)	12.4 (±3.7)
Coma duration (days, mean/sd)	15.5 (±11.5)
Time interval from injury to admission in rehabilitation (days, mean/sd)	69.3 (±82.9)
DRS at admission (median/IQR)	15 (14–17)
LCF at admission (median/IQR)	5 (5–6)
GCS at admission (median/IQR)	8 (8–8)
*Etiology (N/%)*	
Traumatic Brain Injury (TBI)	26 (61.9%)
Vascular	14 (33.3%)
Other etiology	2 (4.8%)

**Table 2:** Kuder–Richardson coefficient of reliability.

Rater	Key Symptoms	Obs.	Item Difficulty	Item Variance	Item-Rest Correlation
A	Cognitive impairment	42	0.86	0.12	0.26
B	Cognitive impairment	42	0.69	0.21	0.26
A2	Cognitive impairment	42	0.81	0.15	0.27
A	Disorientation	42	0.88	0.10	0.37
B	Disorientation	42	0.88	0.10	0.37
A2	Disorientation	42	0.74	0.19	0.29
A	Restlessness	42	0.88	0.10	0.37
B	Restlessness	42	0.90	0.09	0.38
A2	Restlessness	42	0.88	0.10	0.35
A	Fluctuation of presentation	42	0.76	0.18	0.43
B	Fluctuation of presentation	42	0.81	0.15	0.42
A2	Fluctuation of presentation	42	0.69	0.21	0.28
A	Night-time sleep disturbance	42	0.43	0.24	0.55
B	Night-time sleep disturbance	42	0.43	0.24	0.49
A2	Night-time sleep disturbance	42	0.40	0.24	0.60
A	Daytime decreased arousal	42	0.24	0.18	0.52
B	Daytime decreased arousal	42	0.26	0.19	0.54
A2	Daytime decreased arousal	42	0.24	0.18	0.54
A	Psychotic-type symptoms	42	0.38	0.24	0.34
B	Psychotic-type symptoms	42	0.48	0.25	0.38
A2	Psychotic-type symptoms	42	0.40	0.24	0.32
	KR20 coefficient is 0.84				

**Table 3:** Cohen’s k for the inter-rater reliability and intra-rater reliability.

	Inter-Rater Reliability			Intra-Rater Reliability		
Key Symptoms	k	z	*p*	k	z	*p*
Cognitive impairment	0.41	3.00	0.0014	0.49	3.21	0.0007
Disorientation	0.77	5.01	0.0000	0.55	4.00	0.0000
Restlessness	0.63	4.10	0.0000	0.55	3.54	0.0002
Fluctuation of presentation	0.72	4.70	0.0000	0.70	4.63	0.0000
Night-time sleep disturbance	0.90	5.85	0.0000	0.66	4.27	0.0000
Daytime decreased arousal	0.94	6.08	0.0000	0.74	4.78	0.0000
Psychotic-type symptoms	0.81	5.33	0.0000	0.75	4.87	0.0000

**Table 4:** Spearman’s rho rank correlation coefficient.

	A CAP Score	B CAP Score	A2 CAP Score	LCF	DRS
A CAP score	1.00				
B CAP score	0.90 *	1.00			
A2 CAP score	0.64 *	0.71 *	1.00		
LCF	−0.46 *	−0.55 *	−0.47 *	1.00	
DRS	0.22	0.28	0.33 *	−0.70 *	1.00

* *p* < 0.05.

**Table 5:** Cramer’s V for each couple of key symptoms.

	CI	D	R	FOP	NSD	DDA	PS
CI	1.00						
D	0.32	1.00					
R	0.14	0.25	1.00				
FOP	0.13	0.29	0.22	1.00			
NSD	0.09	0.14	0.21	0.12	1.00		
DDA	0.18	0.07	0.19	0.13	0.46	1.00	
PS	0.11	0.18	0.17	0.17	0.37	0.23	1.00

**Legend:** CI (cognitive impairment), D (disorientation, R (restlessness), FOP (fluctuation of presentation), NSD (night-time sleep disturbance), DDA (daytime decreased arousal), PS (psychotic-type symptoms).

**Table 6:** Percentages of key symptoms (and IC95%) by LCF groups.

	LCF 4–5	LCF 6	*p*
Cognitive impairment	91.7% *(73.0–99.0%)*	70.0% *(34.7–93.3%)*	0.104
Disorientation	83.3% *(62.6–95.3%)*	40.0% *(12.1–73.8%)*	0.012
Restlessness	95.8% *(78.9–99.9%)*	80.0% *(44.4–97.5%)*	0.138
Fluctuation of presentation	83.3% *(62.6–95.3%)*	70.0% *(34.7–93.3%)*	0.381
Night-time sleep disturbance	45.8% *(25.6–67.2%)*	30.0% *(6.7–65.2%)*	0.393
Daytime decreased arousal	33.3% *(15.6–55.3%)*	0.0% *(0.0% 30.8%)*	0.037
Psychotic-type symptoms	37.5% *(18.8–59.4%)*	40.0% *(12.1–73.8%)*	0.891

**Table 7:** Total CAP score vs. LCF scale.

CAP Score	LCF 4	LCF 5	LCF 6	Total
**N** **ot confused**	1	3	11	14
**Mild**	2	36	20	58
**Moderate**	5	13	7	25
**Severe**	16	11	2	29
**Total**	24	63	39	126

## Data Availability

The data presented in this study are available on request from the corresponding author. The data are not publicly available in order to respect participants’ privacy.
